# Supplementary material for: Intertransverse process block to improve quality of recovery and pain management in cardiac surgery: Protocol for a double-blinded randomized controlled trial
Source: PLoS One. 2025 Sep 24;20(9):e0328954. doi: 10.1371/journal.pone.0328954 (PMC12459764; doi:10.1371/journal.pone.0328954)
Supplement: S2 File — (PDF) [file pone.0328954.s002.pdf]

Intertransverse Process Block for Chronic Postsurgical Pain in Adult Cardiac Surgical Patients: Protocol for a Prospective Double-Blinded Randomized Controlled Trial

***Corresponding author:** Dr Henry MK Wong, Department of Anaesthesia and Intensive Care, Prince of Wales Hospital. 30-32 Ngan Shing Street, Shatin, New Territories, Hong Kong (email: wmk047@ha.org.hk)

Competing interest: None declared

Date and protocol version: 4 April 2025, Version 2.0

Introduction

The cornerstone of modern pain management in cardiac surgery lies in proactively preventing the development of chronic postsurgical pain (CPSP). This paradigm shift emphasizes pre-emptive interventions to disrupt the cascade of events and central sensitization that transform acute postoperative pain into a persistent, debilitating condition.[1-4] CPSP, a significant and often underestimated consequence of cardiac surgery, affects a substantial proportion of patients, with prevalence rates ranging from 28% to 56% in the years following surgery.[5-8] Its impact extends beyond mere discomfort, significantly impairing daily function, reducing quality of life,[8-9] and imposing a substantial economic burden on the healthcare system.

The pathophysiology of CPSP involves a complex interplay of factors, with central sensitization playing a pivotal role.[3] This process, characterized by heightened neuronal excitability within the central nervous system, amplifies pain signals and can lead to persistent pain even after the initial injury has healed. Therefore, a crucial aspect of CPSP prevention lies in disrupting the afferent nociceptive signals transmitted from the injured tissues to the spinal cord and brain, thereby preventing the establishment and perpetuation of central sensitization.[1,4]

While opioids have traditionally been the mainstay of postoperative pain management, their use is not without significant drawbacks. Opioids can have dose-dependent side effects, including respiratory depression, nausea, and constipation. Furthermore, prolonged opioid use can lead to tolerance, opioid-induced hyperalgesia, and an increased risk of chronic opioid use.[10-11] Recognizing these limitations, the current emphasis in pain management is on multimodal analgesia strategies that incorporate non-opioid medications and regional anaesthesia techniques to optimize pain control while minimizing the risks associated with opioid use.

Non-steroidal anti-inflammatory drugs (NSAIDs) offer a valuable adjunct in pain management, but their use in cardiac surgery is often limited due to concerns regarding bleeding complications and potential renal impairment. Other non-opioid analgesics, such as paracetamol and gabapentinoids, have shown limited efficacy in managing the intense pain associated with sternotomy.[13] Regional anaesthesia techniques have emerged as promising

strategies for both acute pain management and potential CPSP prevention in various surgical settings.[14-17] Techniques such as epidural anaesthesia and paravertebral blocks have demonstrated efficacy in reducing postoperative pain intensity and opioid requirements.[14-15] However, their application in cardiac surgery presents unique challenges. Epidural anaesthesia carries the risk of neuraxial hematoma due to systemic anticoagulation and heparinization, while paravertebral blocks may be associated with complications such as pneumothorax and pleural puncture.[18] Erector spinae plane block (ESPB) has shown inconsistent results in reducing postoperative pain and morphine consumption in cardiac surgery.[19-24] Parasternal plane blocks offer advantages over neuraxial techniques[25-26] but may not adequately address visceral pain.

While regional anaesthesia is crucial for managing acute postoperative pain, its impact on CPSP remains largely unknown. The potential for regional anaesthesia to reduce CPSP has been identified as one of the top research priorities in anaesthesia and perioperative care.[27] Intertransverse process block (ITPB) is a novel and promising alternative, targeting the paravertebral space through extra-paravertebral injection within the intertransverse tissue complex, posterior to the superior costotransverse ligament (SCTL).[28-29] Recent MRI studies have demonstrated consistent spread of local anaesthetic to the ipsilateral intercostal, paravertebral spaces, neural foramina, and epidural space following ITPB,[30] suggesting potential for both somatic and visceral analgesia. It demonstrated preferential spread of LA to the epidural space and neural foramina over the ESPB, and into the thoracic paravertebral space with effective analgesia after breast and video-assisted thoracoscopic surgeries.[31-32] Compared to other regional techniques in cardiac surgery, ITPB may offer a simpler and safer approach with reduced risk of pleural puncture and bleeding. Therefore, this trial will assess the analgesic efficacy of ITPB aimed at mitigating both acute and chronic postsurgical pain in cardiac surgical patients. We hypothesis ITPB will reduce CPSP after cardiac surgery, improve pain control, improve quality of recovery and health-related quality of life. The primary aim is to investigate the efficacy of intertransverse process block (ITPB) on CPSP after cardiac surgery by determining the incidence of CPSP at 3-month, defined as persistent pain that was not present before surgery or that had different characteristics, and other possible causes of pain are excluded. The secondary aims are to investigate the quality of recovery after cardiac surgery, and its analgesic efficacy in the immediate postoperative period, and to investigate the efficacy of ITPB on the incidence of CPSP at 6-month and 12-month, and the pain interference (sensory and affective components, physical activities) at 3-month, 6-month and 12-month after surgery.

Methods

This is a prospective, parallel, double-blinded, randomized controlled trial. Patients undergoing elective cardiac surgery in Prince of Wales Hospital, a university teaching hospital with 1650 beds in Hong Kong, are eligible for enrolment. All patients will be admitted to a 23-bed ICU for early postoperative care and monitored with 1:1 nursing at all times, with an expectation of discharge from ICU to a high-dependency cardiac ward within 24 hours after surgery. Currently, 350-400 adults undergo elective coronary artery bypass graft (CABG) and/or valvular surgery each year.

Inclusion: Adult patients aged 18 or older, undergoing elective CABG, valve repair/replacement, or combined CABG/valve procedure via sternotomy, will be included.

Exclusion: Emergency surgery, redo surgery, history of chronic pain or being on chronic opioids/sedatives, renal failure with an estimated glomerular filtration rate ≤30 ml/min (calculated by Cockcroft-Gault formula), re-operation within 24 hours after surgery, intraoperative use of remifentanil, and inability to provide informed consent.

Randomization and Concealment

Patients are randomly allocated to interventional or control group by drawing sequentially numbered, coded, sealed, opaque envelopes, each containing the group assignment of either interventional or control. The sealed envelopes for randomization will be prepared by a third party who takes no further part in the study. The ITPB syringes are prepared under strict asepsis by a nurse not involved in the study. ITPB is performed by a single anaesthesiologist who is blinded to group allocation. The primary care team, blinded to group allocation, performs surgeries using standardized technique. Anaesthesiologists and nurses blinded to group allocation recorded data intraoperatively, in the ICU, and at regular intervals in cardiac wards. Data will be recorded at 2-hour, 4-hour, 8-hour, 12-hour, 24-hour, 48-hour and 72-hour after extubation and at 1-month, 3-month, 6-month and 12 months after surgery by blinded personnel.

Anaesthesia and Interventions

All patients receive standard cardiac surgery monitoring. General anaesthesia is induced with midazolam 0.01-0.05 mg/kg, fentanyl 2-5 mcg/kg, and rocuronium 0.5-1 mg/kg to facilitate intubation. Anaesthesia is maintained with sevoflurane and propofol infusion, targeting a Bispectral Index of 40-60. ITPB is performed after anaesthesia induction with the patient in lateral decubitus positioning. Intraoperative opioids (fentanyl and morphine) will be administered at the discretion of the anaesthesiologist. The postoperative analgesia protocol was identical in both study groups, including patient-controlled analgesia (PCA) morphine protocol for 72 hours after surgery, oral analgesics (paracetamol 1g every 6 hours, dihydrocodeine 30mg three times a day), and on-demand antiemetics (intravenous ondansetron 4mg every 8 hours). Rescue analgesics on top of the protocol regimen can be prescribed as needed. Upon ICU admission, propofol infusion is stopped to facilitate weaning from ventilator using Adaptive Support Ventilation (ASV), which adjusts the ventilation parameters depending on the patent's lung mechanics and effort. Pain will be assessed regularly in ICU and on the ward. Upon extubation, pain scores are assessed at 2-hour, 4-hour, 8-hour, 12-hour, 24-hour, 48-hour and 72-hour. Patients receive PCA morphine for moderate to severe pain. Nausea, vomiting and rescue antiemetics are documented.

Ultrasound Block Placement

The intervention group received bilateral ITPB after GA induction, whilst sham blocks are performed in the control group. All blocks are performed by an anaesthesiologist who had previously performed ≥50 successful ITPB blocks, using Philips EPIQ ultrasound system, with a curved array transducer (C5-1), and 80mm echogenic nerve block needle (SonoTAP; PAJUNK, Germany). ITPB is performed with the patients positioned in a lateral decubitus position. The target intervertebral level (T4-5) is identified and marked in the preview ultrasound scan. The transducer is placed 2-3 cm lateral to the spinous process. Under strict asepsis, a single-level (T4-5) ultrasound-guided ITPB is performed with the in-plane insertion of the block needle from lateral to medial direction until its tip is at the medial aspect of the retro-SCTL space. After confirming the needle position by distension of the retro-SCTL space after a test bolus injection of 1-2 ml 0.9% normal saline, 25 ml 0.25% levobupivacaine or placebo is injected via the nerve block needle in small aliquots. The same procedure is repeated on the other side with the same volume of study medication. The time required to perform the block will be recorded as the time from insertion of block needle to removal from the patient after injection.

Outcome measures

Though acute pain is identified as an important predictor for the development of CPSP, advanced pain management strategies have failed to reduce the overall incidence of CPSP, suggesting a complex underlying mechanism. Evidence has demonstrated that pain-related functional interference and patient-reported outcomes, such as quality of recovery, might be associated with the development of CPSP.[33] Thus, the primary outcome of this study is the Quality of Recovery (QoR-15) score at 24 hours after extubation. QoR is recommended for patient's comfort after surgery, and is highly valid and reliable patient-centred outcome measure.[34]

Secondary outcomes include Numerical Rating Scale (NRS) pain scores at 2-hour, 4-hour, 8-hour, 12-hour, 24-hour, 48-hour and 72-hour after extubation, patient satisfaction with pain management and postoperative morphine consumption at the above time points, the time for first morphine rescue (in minutes), intraoperative opioids consumption (converted into morphine equivalence), duration of mechanical ventilation, length of stay in ICU and hospital, side effects associated with opioids such as postoperative nausea and vomiting (PONV), incidence of CPSP at 3-month, 6-month and 12-month, and pain interference measured with Short-Form McGill Pain Questionnaire-2 (SF-MPQ-2) and Brief Pain Inventory (BPI) Interference Scale at 3-month, 6-month and 12-month after surgery.

CPSP is defined as persistent pain after surgery that was not present before surgery or that had different characteristics, and other possible causes of pain are excluded. Participants reported CPSP will further be assessed on the severity and impact of chronic pain based on the recommendations by the Initiative on Methods, Measurement, and Pain Assessment in Clinical Trials (IMMPACT), including the SF-MPQ-2 to assess the sensory pain qualities and affective components, and the BPI to assess the interference of pain with physical functioning.

Chinese version SF-MPQ-2[35] is used to measure the sensory and affective aspects of pain. It evaluates symptoms related to chronic pain on a 11-point numerical rating scale (0 = none, 10 = worst possible). There are three sensory descriptors and one affective descriptor. The four subscales will be calculated as a mean of items in each subscale, and the total score is calculated as the mean of all 22 items. Higher subscale or total scale scores indicate more intense symptoms. The Chinese version of the Brief Pain Inventory (BPI) Interference Scale[36] is used

to evaluate the extent of pain interfering with various components of functioning, including physical functioning, emotional functioning and sleep.

Data collection (Appendix A)

After screening for eligibility, patients will be given information sheets regarding the main aspects of the trial and information will be discussed with the research nurse before informed consent is obtained. All data is collected by research team members blinded to group assignment. Patient demographics and body mass index are recorded. Cumulated opioid consumption data and time to first morphine rescue are extracted from PCA pump. At 2-hour, 4-hour, 8-hour, 12-hour, 24-hour, 48-hour and 72-hour post-extubation, pain scores at rest and on coughing are quantified using NRS from 0 to 10. Zero represents no pain at all while 10 points represents the worst pain ever. The patients are asked to rate the overall satisfaction to pain management on a verbal analogue scale (0=worst possible, 100=best possible) at the predefined points. Any nausea and vomiting, and use of rescue antiemetics are documented. The Chinese validated QoR-15[37] will be completed at baseline (preoperatively) and postoperatively at 24-hour and 72-hour after extubation. The SF-MPQ-2 and Brief Pain Inventory will be used to evaluate the CPSP at 3-month, 6-month and 12-month after surgery.

Statistics and sample size calculation

Sample size was calculated using G*Power software version 3.1.9.3 (Kiel University, Kiel, Germany), based on the QoR-15 score at 24 hours postoperatively — the primary outcome. The minimum clinically important difference (MCID) for the QoR-15 score is eight points,[38] and the typical standard deviation (SD) ranges from 10 to 16.[39-40] Assuming a two-sided type I error of 0.05, type II error of 0.2, and a population variance of 144 (SD = 12), a sample size of 36 per group is required. Allowing for a 20% dropout rate as a result of loss to follow up after patients discharge, a total of 96 patients (48 patients per group) will provide 80% power to detect a mean difference of ≥8 points in the QoR-15 score at 24 hours between the two groups.

All outcomes will be analyzed and reported on an intention-to-treat basis, with patients analyzed according to their randomized group regardless of protocol adherence. A secondary per protocol analysis will be conducted for patients who do not adhere fully to the study protocol. Given the repeated measures of pain scores over time, which are correlated,

Generalised Estimating Equation (GEE) models will be used to assess the time effects of postoperative analgesia. Categorical data will be reported as counts and percentages. Continuous variables will be presented as mean (standard deviation) or median (interquartile range), depending on normality assessed using the Shapiro-Wilk's test. Between-group comparison will be conducted using the independent sample *t*-test for parametric data and Mann-Whitney U test for nonparametric data. Categorical variables will be compared using the Chi-square test. Data analyses will be performed using SPSS 27.0 (IBM Corp, Armonk, NY), and GEE modelling will be conducted using Stata V.14 (Statam College Station, Texas, USA), with a Gaussian distribution, identify-link function, exchangeable correlation structure, and robust standard errors. A P-value of <0.05 will be considered statistically significant, without adjusting for multiple comparisons.

Ethics, data management and dissemination

Patients will be screened for recruitment on the day prior to surgery, and the risks and benefits of the study will be explained. Written informed consent will be obtained from the patient. Patients may withdraw from the project at any time without prejudice. Study participants will be assigned a unique code as identifier throughout the study. All data will be entered into an electronic system by research team members who are trained in data entry. To ensure accurate data entry, a second member of the research team will check data entry. Data collection and study conduct will be monitored with the research team on weekly basis to ensure protocols are implemented consistently. All data will be kept confidential and maintained on a password-protected computer and in locked filing cabinets within the secure offices of the Department of Anesthesia and Intensive Care. Digital files will be securely deleted, and paper documents shredded, after 5 years. Only group data will be published. Access to data will be restricted to study investigators. Approval for the project has been obtained from The Joint Chinese University of Hong Kong-New Territories East Cluster Clinical Research Ethics Committee. The study will adhere to local laws, Declaration of Helsinki, International Council for Harmonization of Technical Requirements for Pharmaceuticals for Human Use Good Clinical Practice and Institutional Policies. All adverse events associated with the study drug will be recorded by the research team and reported to the trial management committee. The trial management committee, comprising external and independent clinicians, will review all events within 48 hours and discuss them at regular trial committee meetings. The results of this study will be disseminated at international conferences and in peer-reviewed journals.

REFERENCES

1. Katz J, Clarke H, Seltzer Z. Review article: Preventive analgesia: quo vadimus? Anesth Analg. 2011 Nov;113(5):1242-53. doi: 10.1213/ANE.0b013e31822c9a59. Epub 2011 Sep 30. Erratum in: Anesth Analg. 2011 Dec;113(6):1475.
2. Ong CK, Lirk P, Seymour RA, Jenkins BJ. The efficacy of preemptive analgesia for acute postoperative pain management: a meta-analysis. Anesth Analg. 2005 Mar;100(3):757-773. doi: 10.1213/01.ANE.0000144428.98767.0E.
3. Woolf CJ, Chong MS. Preemptive analgesia--treating postoperative pain by preventing the establishment of central sensitization. Anesth Analg. 1993 Aug;77(2):362-79. doi: 10.1213/00000539-199377020-00026.
4. Sandkühler J, Gruber-Schoffnegger D. Hyperalgesia by synaptic long-term potentiation (LTP): an update. Curr Opin Pharmacol. 2012 Feb;12(1):18-27. doi: 10.1016/j.coph.2011.10.018.
5. Eisenberg E, Pultorak Y, Pud D, Bar-El Y. Prevalence and characteristics of post coronary artery bypass graft surgery pain (PCP). Pain. 2001 May;92(1-2):11-7. doi: 10.1016/s0304-3959(00)00466-8.
6. Kalso E, Mennander S, Tasmuth T, Nilsson E. Chronic post-sternotomy pain. Acta Anaesthesiol Scand. 2001 Sep;45(8):935-9. doi: 10.1034/j.1399-6576.2001.450803.x.
7. Meyerson J, Thelin S, Gordh T, Karlsten R. The incidence of chronic post-sternotomy pain after cardiac surgery--a prospective study. Acta Anaesthesiol Scand. 2001 Sep;45(8):940-4. doi: 10.1034/j.1399-6576.2001.450804.x.
8. Gjeilo KH, Stenseth R, Wahba A, Lydersen S, Klepstad P. Chronic postsurgical pain in patients 5 years after cardiac surgery: A prospective cohort study. Eur J Pain. 2017 Mar;21(3):425-433. doi: 10.1002/ejp.918.
9. Choinière M, Watt-Watson J, Victor JC, Baskett RJ, Bussières JS, Carrier M, Cogan J, Costello J, Feindel C, Guertin MC, Racine M, Taillefer MC. Prevalence of and risk factors for persistent postoperative nonanginal pain after cardiac surgery: a 2-year prospective multicentre study. CMAJ. 2014 Apr 15;186(7):E213-23. doi: 10.1503/cmaj.131012.
10. Gutwinski, S., Schoofs, N., Stuke, H., et al. Opioid tolerance in methadone maintenance treatment: comparison of methadone and levomethadone in long-term treatment. *Harm Reduct J* **13**, 7 (2016). https://doi.org/10.1186/s12954-016-0095-0
11. Hah JM, Bateman BT, Ratliff J, Curtin C, Sun E. Chronic Opioid Use After Surgery: Implications for Perioperative Management in the Face of the Opioid Epidemic. Anesth Analg. 2017 Nov;125(5):1733-1740. doi: 10.1213/ANE.0000000000002458.
12. Kehlet H, Jensen TS, Woolf CJ. Persistent postsurgical pain: risk factors and prevention. Lancet. 2006 May 13;367(9522):1618-25. doi: 10.1016/S0140-6736(06)68700-X.
13. Verret M, Lauzier F, Zarychanski R, Perron C, Savard X, Pinard AM, Leblanc G, Cossi MJ, Neveu X, Turgeon AF; Canadian Perioperative Anesthesia Clinical Trials (PACT) Group. Perioperative Use of Gabapentinoids for the Management of Postoperative Acute Pain: A Systematic Review and Meta-analysis. Anesthesiology. 2020 Aug;133(2):265-279. doi: 10.1097/ALN.0000000000003428.

14. Zhou K, Li D, Song G. Comparison of regional anesthetic techniques for postoperative analgesia after adult cardiac surgery: bayesian network meta-analysis. Front Cardiovasc Med. 2023 May 22;10:1078756. doi: 10.3389/fcvm.2023.1078756.
15. Naganuma M, Tokita T, Sato Y, et al. Efficacy of Preoperative Bilateral Thoracic Paravertebral Block in Cardiac Surgery Requiring Full Heparinization: A Propensity-Matched Study. J Cardiothorac Vasc Anesth. 2022 Feb;36(2):477-482. doi: 10.1053/j.jvca.2021.05.001
16. Andreae MH, Andreae DA. Regional anaesthesia to prevent chronic pain after surgery: a Cochrane systematic review and meta-analysis. Br J Anaesth. 2013 Nov;111(5):711-20. doi: 10.1093/bja/aet213.
17. Kukreja, P., Paul, L.M., Sellers, A.R. *et al.* The Role of Regional Anesthesia in the Development of Chronic Pain: a Review of Literature. *Curr Anesthesiol Rep* **12**, 417–438 (2022). https://doi.org/10.1007/s40140-022-00536-y
18. Richardson J, Lönnqvist PA, Naja Z. Bilateral thoracic paravertebral block: potential and practice. Br J Anaesth. 2011 Feb;106(2):164-71. doi: 10.1093/bja/aeq378.
19. Wang W, Yang W, Liu A, Liu J, Yuan C. The Analgesic Effect of Ultrasound-guided Erector Spinae Plane Block in Median Sternotomy Cardiac Surgery in Adults: A Systematic Review and Meta-analysis of Randomized Controlled Trials. J Cardiothorac Vasc Anesth. 2024 Nov;38(11):2792-2800. doi: 10.1053/j.jvca.2024.05.019.
20. Macaire P, Ho N, Nguyen T, et al. Ultrasound-Guided Continuous Thoracic Erector Spinae Plane Block Within an Enhanced Recovery Program Is Associated with Decreased Opioid Consumption and Improved Patient Postoperative Rehabilitation After Open Cardiac Surgery-A Patient-Matched, Controlled Before-and-After Study. J Cardiothorac Vasc Anesth. 2019 Jun;33(6):1659-1667. doi: 10.1053/j.jvca.2018.11.021.
21. Dost, B., De Cassai, A., Balzani, E. et al. Effects of ultrasound-guided regional anesthesia in cardiac surgery: a systematic review and network meta-analysis. BMC Anesthesiol **22**, 409 (2022). https://doi.org/10.1186/s12871-022-01952-7
22. Karmakar MK, Lönnqvist PA. The clinical use of the thoracic erector spinae plane block. Con - ESPB is not useful for thoracic analgesia. J Clin Anesth. 2024 May;93:111353. doi: 10.1016/j.jclinane.2023.111353
23. Oostvogels L, Weibel S, Meißner M, et al. Erector spinae plane block for postoperative pain. Cochrane Database Syst Rev. 2024 Feb 12;2(2):CD013763. doi: 10.1002/14651858.CD013763.pub3.
24. Sørenstua M, Zantalis N, Raeder J, et al. Spread of local anesthetics after erector spinae plane block: an MRI study in healthy volunteers. Reg Anesth Pain Med. 2023 Feb;48(2):74-79. doi: 10.1136/rapm-2022-104012.
25. Wong HMK, Chen PY, Tang GCC, et al. Deep Parasternal Intercostal Plane Block for Intraoperative Pain Control in Cardiac Surgical Patients for Sternotomy: A Prospective Randomized Controlled Trial. J Cardiothorac Vasc Anesth. 2024 Mar;38(3):683-690. doi: 10.1053/j.jvca.2023.11.038.
26. Capuano P, Sepolvere G, Toscano A, et al. Fascial plane blocks for cardiothoracic surgery: a narrative review. J Anesth Analg Crit Care. 2024 Mar 11;4(1):20. doi: 10.1186/s44158-024-00155-5.

27. Lewis O, Lloyd J, Ferry J, Macfarlane AJR, Womack J, El-Boghdadly K, Shelton CL, Schaff O, Quick TJ, Smith AF, Cannons K, Pearson A, Heelas L, Rodger D, Marshall J, Pellowe C, Bowness JS, Kearns RJ. Regional anaesthesia research priorities: a Regional Anaesthesia UK (RA-UK) priority setting partnership involving patients, carers and healthcare professionals. Anaesthesia. 2025 Feb;80(2):170-178. doi: 10.1111/anae.16473.
28. Bowness JS, Pawa A, Turbitt L, et al. International consensus on anatomical structures to identify on ultrasound for the performance of basic blocks in ultrasound-guided regional anesthesia. Reg Anesth Pain Med. 2022 Feb;47(2):106-112. doi: 10.1136/rapm-2021-103004.
29. Costache I, de Neumann L, Ramnanan CJ, Goodwin SL, Pawa A, Abdallah FW, McCartney CJL. The mid-point transverse process to pleura (MTP) block: a new end-point for thoracic paravertebral block. Anaesthesia. 2017 Oct;72(10):1230-1236. doi: 10.1111/anae.14004.
30. Pangthipampai P, Siriwanarangsun P, Pakpirom J, Sivakumar RK, Karmakar MK. Intertransverse process block (ITPB) at the retro-superior costotransverse ligament (retro-SCTL) space: Evaluation of local anesthetic spread using MRI and sensory blockade in healthy volunteers. J Clin Anesth. 2024 Dec 18;101:111718. doi: 10.1016/j.jclinane.2024.111718.
31. Bhoi D, Narasimhan P, Nethaji R, Talawar P. Ultrasound-Guided Midpoint Transverse Process to Pleura Block in Breast Cancer Surgery: A Case Report. A A Pract. 2019 Feb 1;12(3):73-76. doi: 10.1213/XAA.0000000000000850.
32. Karmakar MK, Sivakumar RK, Sheah K, Pangthipampai P, Lönnqvist PA. The Retro Superior Costotransverse Ligament Space as a New Target for Ultrasound-Guided Intertransverse Process Block: A Report of 2 Cases. A A Pract. 2022 Jul 22;16(7):e01610. doi: 10.1213/XAA.0000000000001610.
33. Maurice-Szamburski A, Bringuier S, Auquier P, Capdevila X. From pain level to pain experience: redefining acute pain assessment to enhance understanding of chronic postsurgical pain. Br J Anaesth. 2024 Nov;133(5):1021-1027. doi: 10.1016/j.bja.2024.08.003.
34. Stark PA, Myles PS, Burke JA. Development and psychometric evaluation of a postoperative quality of recovery score: the QoR-15. Anesthesiology. 2013 Jun;118(6):1332-40. doi: 10.1097/ALN.0b013e318289b84b.
35. A validation study of the Cantonese Chinese version of short form McGill pain questionnaire 2 in Cantonese-speaking patients with chronic pain in Hong Kong
36. Ger LP, Ho ST, Sun WZ, Wang MS, Cleeland CS. Validation of the Brief Pain Inventory in a Taiwanese population. J Pain Symptom Manage. 1999 Nov;18(5):316-22. doi: 10.1016/s0885-3924(99)00087-1.
37. Bu XS, Zhang J, Zuo YX. Validation of the Chinese Version of the Quality of Recovery-15 Score and Its Comparison with the Post-Operative Quality Recovery Scale. Patient. 2016 Jun;9(3):251-9. doi: 10.1007/s40271-015-0148-6.
38. Myles PS, Myles DB, Galagher W, Chew C, MacDonald N, Dennis A. Minimal Clinically Important Difference for Three Quality of Recovery Scales. Anesthesiology. 2016;125:39–45.
39. Moorthy A, Ní Eochagáin A, Dempsey E, Wall V, Marsh H, Murphy T, et al. Postoperative recovery with continuous erector spinae plane block or video-assisted paravertebral block

after minimally invasive thoracic surgery: a prospective, randomised controlled trial. Br J Anaesth. 2023;130:e137–47.

40. Zhang H, Qu Z, Miao Y, Zhang Y, Qian L, Hua B, et al. Comparison between ultrasound-guided multi-injection intertransverse process and thoracic paravertebral blocks for major breast cancer surgery: a randomized non-inferiority trial. Reg Anesth Pain Med. 2023;48:161–6

Appendix A

The following medical and surgical data during the hospital stays will be extracted from electronic record:

1. Patient demographics (age, gender, EuroScore)
2. Type of surgery
3. Duration of surgery and duration of cardiopulmonary bypass
4. ASV time to spontaneous breathing
5. Episodes of nausea and vomiting, and use of rescue antiemetics
6. Length of ICU and hospital stay

		**STUDY PERIOD**									
	**Enrolment**	**Post-allocation**									
TIMEPOINTS	**Baseline**	**2h extubate**	**4h extubate**	**6h extubate**	**8h extubate**	**12h extubate**	**24h extubate**	**48h extubate**	**72h extubate**	**3-month**	**6-month**
**ENROLMENT:**											
**Eligibility screen**	X										
**Informed consent**	X										
**Demographic data**	X										
**Comorbidity data**	X										
**EuroScore**	X										
**Allocation**	X										
**INTERVENTION**											
**ITPB**	X										
**OUTCOMES:**											
**Presence of CPSP**										X	X
**SF-MPQ-2**										X	X

**BPI**										X	X
**QoR-15**	X						X		X		
**Intraoperative morphine equivalent**		X									
**NRS score**		X	X	X	X	X	X	X	X		
**ASV time to spontaneous breathing**		X									
**Time to first morphine rescue**		X	X	X	X	X	X	X	X		
**Postoperative morphine consumption**		X	X	X	X	X	X	X	X		
**Patient satisfaction**		X	X	X	X	X	X	X	X		
**Use of rescue antiemetics**		X	X	X	X	X	X	X	X		
**Nausea/vomiting**		X	X	X	X	X	X	X	X		
**ICU and hospital stay**										X	

Appendix A Assessments overview. NRS, numerical rating scale; ASV, adaptive support ventilation; QoR-15, Quality of Recovery questionnaire; BPI, Brief Pain Inventory; SF-MPQ-2, Short-Form McGill Pain Questionnaire
