# Supplementary material for: Intertransverse process block to improve quality of recovery and pain management in cardiac surgery: Protocol for a double-blinded randomized controlled trial
Source: PLoS One. 2025 Sep 24;20(9):e0328954. doi: 10.1371/journal.pone.0328954 (PMC12459764; doi:10.1371/journal.pone.0328954)
Supplement: Attachment — Submitted filename: Response to Reviewers.docx [file pone.0328954.s004.docx]

To: **Editorial team at *PLOS ONE***

RE: **“Intertransverse process block for chronic postsurgical pain in cardiac surgery: protocol for a double-blinded randomized controlled trial”**

We are pleased to submit a revised copy of our manuscript, for consideration of publication in *PLOS ONE*. The manuscript has been revised to conform to the Journal formatting and style. We thank the reviewers for their insightful and thoughtful commentary. Please find a point-by-point reply for each of their comments:

**Journal Requirements**

1. Please ensure that your manuscript meets PLOS ONE's style requirements, including those for file naming.

Thanks. The manuscript has been revised to conform to PLOS ONE’s formatting and style requirements. The file naming for the supporting information has been revised and uploaded.

2. When completing the data availability statement of the submission form, you indicated that you will make your data available on acceptance. We strongly recommend all authors decide on a data sharing plan before acceptance, as the process can be lengthy and hold up publication timelines. Please note that, though access restrictions are acceptable now, your entire data will need to be made freely accessible if your manuscript is accepted for publication. This policy applies to all data except where public deposition would breach compliance with the protocol approved by your research ethics board. If you are unable to adhere to our open data policy, please kindly revise your statement to explain your reasoning and we will seek the editor's input on an exemption. Please be assured that, once you have provided your new statement, the assessment of your exemption will not hold up the peer review process.

Thanks. Our data will be made available for sharing on a repository upon request, unless for sensitive and confidential patients’ information that will be replaced with patient identifiers.

3. If the reviewer comments include a recommendation to cite specific previously published works, please review and evaluate these publications to determine whether they are relevant and should be cited. There is no requirement to cite these works unless the editor has indicated otherwise.

Thanks. We will carefully review and evaluate those publications for citations as appropriate.

4. Please review your reference list to ensure that it is complete and correct. If you have cited papers that have been retracted, please include the rationale for doing so in the manuscript text, or remove these references and replace them with relevant current references. Any changes to the reference list should be mentioned in the rebuttal letter that accompanies your revised manuscript. If you need to cite a retracted article, indicate the article’s retracted status in the References list and also include a citation and full reference for the retraction notice.

Thanks. The reference list has been reviewed and checked.

**Reviewer 1**

1. In a study investigating the effect of a block on chronic pain, it is not entirely clear why Quality of Recovery (QoR) score was chosen as the primary outcome rather than the incidence of chronic pain itself. Please clarify the rationale and ensure alignment between the study aim and outcome selection

Thanks for the insightful feedback. We appreciate reviewer’s concern regarding the selection of primary outcome measure. Both quality of recovery and chronic postsurgical pain are important patient-related outcomes in cardiac surgery. In the absence of data on analgesic efficacy of the intertransverse process block (ITPB) in cardiac surgery, it is a crucial first step in establishing baseline analgesic data for this novel technique.

We have selected Quality of Recovery-15 (QoR-15) score at 24 hours postoperatively as our primary outcome measure because it is a reliable, validated metric the captures multidimensional patient experience related to postsurgical recovery. The intensity and quality of early recovery are established predictors of long-term outcomes, including the development of chronic postsurgical pain [1]. Superior analgesia and patient comfort in the immediate postoperative period, as captured comprehensively by the QoR-15, are believed to mitigate the central sensitization and neuroplastic changes that underlie the transition from acute to chronic pain. By establishing the efficacy of ITPB in managing acute pain and improving early recovery, the study provides vital preliminary data that can inform the design and power calculations for subsequent larger trials specifically targeting chronic postsurgical pain as the primary outcome.

We have clarified this rationale in the revised manuscript (Lines 457-465) to better highlight the study’s priori role as a foundational investigation for ITPB in cardiac surgery. The title of the manuscript is also revised (Lines 4-6), for both the manuscript and the trial registration, to better align with the aim of the study.

Chronic postsurgical pain outcomes at three months and beyond will be analyzed both with and without adjusting for QoR at baseline and in the immediate postoperative period, to account for potential influence of early recovery on chronic postsurgical pain perception (Lines 365-369).

References:

[1] Maurice-Szamburski A, Bringuier S, Auquier P, Capdevila X. From pain level to pain experience: redefining acute pain assessment to enhance understanding of chronic postsurgical pain. Br J Anaesth. 2024 Nov;133(5):1021-1027. doi: 10.1016/j.bja.2024.08.003

2. I recommend defining chronic postsurgical pain according to the ICD-11 classification.
   Suggested reference:
   Schug SA, Lavand'homme P, Barke A, et al. The IASP classification of chronic pain for ICD-11: chronic postsurgical or posttraumatic pain. Pain. 2019;160(1):45–52. doi:10.1097/j.pain.0000000000001413

Thanks for the recommendation. The definition of chronic postsurgical pain in the manuscript text has been revised (Lines 292-294) with the suggested reference included.

3. The authors should consider and discuss recent studies specifically addressing the impact of ESP block on chronic pain after cardiac surgery, which are highly relevant:
   Dost B, Sarıkaya Ozel E, Kaya C, et al. Incidence of chronic postsurgical pain after cardiac surgery and the effect of bilateral erector spinae plane block: a randomized controlled trial. Reg Anesth Pain Med. Published online May 7, 2025. doi:10.1136/rapm-2025-106591

Also suggested references;
Yu X, Liu C. Intertransverse process block: A narrative review. J Clin Anesth. 2025;104:111857. doi:10.1016/j.jclinane.2025.111857
Dost B, Karapinar YE, Karakaya D, et al. Chronic postsurgical pain after cardiac surgery: A narrative review. Saudi J Anaesth. 2025;19(2):181–189. doi:10.4103/sja.sja_829_24

Thanks for the suggestions. The recent study describing the effect of ESP block on chronic pain after cardiac surgery has been included in the manuscript (Lines 143-144) and appropriately cited. The review article on intertransverse process block is also appropriately cited in the manuscript.

4. The section currently titled “Ultrasound block placement” could be renamed to a more precise heading, for example “Ultrasound-guided block technique”

Thanks for the comment. The section title has been revised as suggested.

**Reviewer 2**

1. Why author choose to do this study single center?

Thank you for the question. A primary reason for doing this study single center is the cost and feasibility. This study is only supported by internal funding from my department. A single center design is therefore significantly less expensive and require fewer resources for administration and coordination, making it a practical choice. There are three cardiac surgical centers in Hong Kong, with slight differences in practice for both surgery and anaesthesia. Moreover, intertransverse process block is a relatively new regional technique that requires training and expertise. By confining the study to one location, we can ensure consistency in clinical practice and staff training. This helps reduce the risk of confounders that can arise from different institutional cultures or techniques, benefiting internal validity, where the observed effects are more likely due to the intervention itself rather than external factors. This is important for a preliminary study without much baseline data from the literature.

We acknowledge the limitation of a single-center design in generalizability. We hope to generate initial findings from this study which supports substantiation to a large-scale study in the future.

2. It should be added who will perform the blocks.

Thank you for the comment. We have indicated in the “Ultrasound-guided block technique” section that the study intervention will be performed by an experienced anesthesiologist who completed more than fifty successful ITPB procedures and takes no further part in the study.

3. Primary and secondary end points should be stated, also because sample size is computed on the primary E.P.

Thank you for the comment. The primary outcome is the Quality of Recovery-15 (QoR-15) score at 24 hours after tracheal extubation. The secondary outcomes include acute pain scores, time to first morphine rescue, patient satisfaction, postoperative morphine requirements, opioid-related side effects, and time to extubate. Chronic pain parameters such as incidence of chronic postsurgical pain, and the pain interferences will be recorded as secondary outcomes.

We have specifically highlighted and clearly stated our primary and secondary outcomes in the manuscript.

4. Blinding should be better detailed.

Thank you for the comments. The blinding details, including preparation of the randomization envelopes, the study syringes, and the personnels involved in the preparation are revised with better details (Lines 210-215)
